# Case Report: Subtotal Lymphoid and Total Marrow Irradiation as Bridge Therapy to CD19-Directed CAR T Cells in a Chemorefractory DLBCL With Leukemic Involvement

**DOI:** 10.3389/fimmu.2022.934700

**Published:** 2022-07-14

**Authors:** Simonetta Saldi, Vincenzo Maria Perriello, Lorenza Falini, Loredana Ruggeri, Christian Fulcheri, Sara Ciardelli, Alessandra Innocente, Stelvio Ballanti, Nicodemo Baffa, Leonardo Flenghi, Antonio Pierini, Cynthia Aristei, Brunangelo Falini

**Affiliations:** ^1^Radiation Oncology Section, Department of Medicine and Surgery, University of Perugia, Perugia, Italy; ^2^Hematology Section, Department of Medicine and Surgery, Center for Hemato-Oncological Research, University of Perugia, Perugia, Italy; ^3^Health Physics Department, Ospedale S. Maria della Misericordia, Perugia, Italy; ^4^Nuclear Medicine, Ospedale S. Maria della Misericordia, Perugia, Italy

**Keywords:** CAR (chimeric antigen receptor) T cells, radiotherapy, diffuse large B-cell lymphoma, bridge therapy, gene therapy

## Abstract

CAR T cell therapy has transformed the salvage approach for relapsed/refractory diffuse large B-cell lymphoma (R/R DLBCL). Maintaining disease control before CAR T cell infusion during product manufacturing (so-called bridging therapy) is an important step to optimizing outcome. Among possible bridging therapies, radiation therapy (RT) represents a valuable option, particularly when the disease is limited. Here, we report for the first time on a patient with chemorefractory-transformed DLBCL showing nodal, extranodal, and massive bone marrow (BM) lymphoma infiltration associated with leukemic involvement, a successful bridge therapy to CD19-directed CAR T cell therapy by subtotal lymphoid/total marrow irradiation plus thiothepa followed by reinfusion of CD34+ autologous hematopoietic stem cells. Such a novel bridging regimen allowed a significant reduction of nodal and BM tumor volume while improving blood cell count before CAR T cell infusion. The PET-CT scan and BM evaluation performed at 1, 3, and 6 months after treatment showed complete remission of the disease. A relapse occurred at almost 1 year in lymph nodes because of CD19 antigen escape while the BM remained free of disease. This extended radiotherapy approach may be an effective bridging therapy for chemorefractory DLBCL patients eligible for CAR T cells who present with a high tumor burden, including massive BM involvement associated with leukemic involvement. This preliminary evidence is worth confirming in additional patients.

## Introduction

Chimeric antigen receptor (CAR) T cells directed against the CD19 B-cell molecule (tisagenlecleucel, axicabtagene, and lisocabtagene) induce long-term complete responses (CRs) in about 40% of relapsed/refractory (R/R) diffuse large B-cell lymphoma (DLBCL) patients (1–3). However, about 60% of cases show no or only temporary response to anti-CD19 CAR T cells because of several factors, including immune escape due to CD19 loss (4) or insufficient CAR T cell expansion/persistence *in vivo* (5).

Another major obstacle to the success of this adoptive T-cell therapy is the inability to control disease progression before CAR T-cell infusion, particularly in patients with very high tumor burden, including massive bone marrow (BM) involvement. Bridging approaches to CAR T cells in chemorefractory DLBCL include polatuzumab vedotin (drug-conjugated anti-CD79b monoclonal antibody)-bendamustine-rituximab (6), drug-conjugated monoclonal antibodies directed against CD19 (7), bispecific antibodies (anti CD3/CD20) (7), or radiotherapy (RT) (8–10).

Here, we report on a 49-year-old woman with nodal, left iliopsoas muscle, BM, and subsequent leukemic involvement by chemorefractory DLBCL who was successfully bridged to CAR T cell therapy using subtotal lymphoid irradiation (sTLI) followed by total marrow irradiation (TMI) plus thiothepa and reinfusion of CD34+ autologous hematopoietic stem cells. To our knowledge, this is the first time sTLI/TMI has been adopted as a bridge therapy to allow the infusion of CAR T cells.

## Case presentation

**Figure 1:**
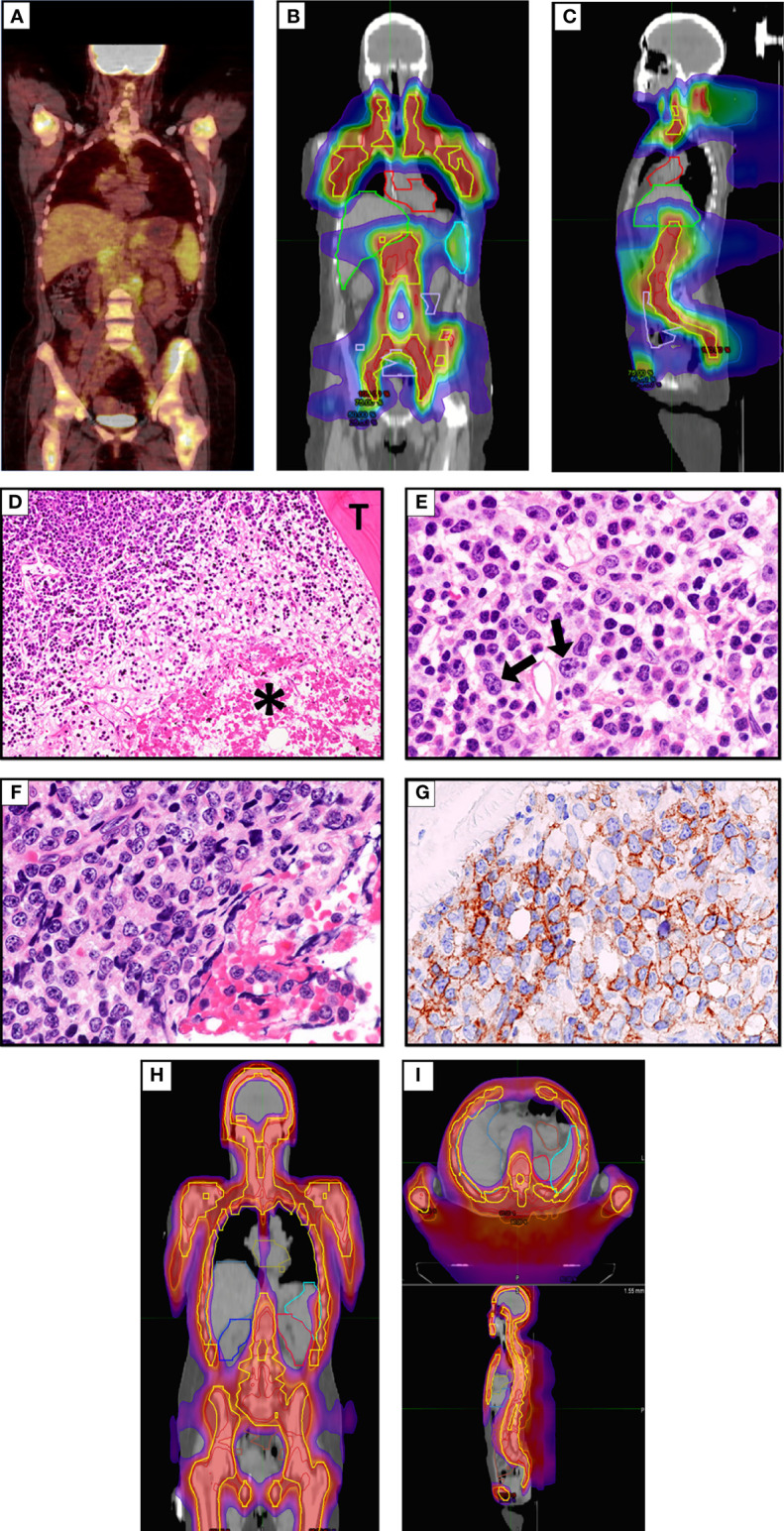
**(A)** FDG-PET/CT coronal maximum intensity projection (MIP) image before sTLI showing avid uptake of BM, multiple lymph nodes and iliopsoas muscle. **(B, C)** sTLI dose distribution color wash **(B)**, coronal view **(C)**, sagittal view. **(D)** Massive BM involvement by DLBCL. The asterisk * indicates a large area of necrosis. T indicates a BM trabecula (Hematoxylin–Eosin; × 100). **(E)** An area from the same section as **(D)** showing infiltration by low grade B-cell lymphoma and occasional large cells (arrows) (Hematoxylin–Eosin; ×400). **(F)** The same section as **(D)** showing another area infiltrated by DLBCL cells (Hematoxylin–Eosin; ×400), that express the CD19 molecule **(G)** (Leica immunoperoxidase staining; ×400). **(H, I)** Total marrow irradiation (TMI) dose distribution color wash **(H)**, coronal view **(I)**, axial and sagittal view.

A 49-year-old woman presented in 2019 because of low back pain, fever, and night sweats. A BM biopsy revealed a massive infiltration by CD5+ DLBCL, probably secondary to low grade B-cell lymphoma not otherwise specified. The FISH analysis showed monoallelic deletion of *TP53* and amplification of the *MYC* gene (range 4–9 signals) in virtually all tumor cells; no rearrangements of *BCL2* and *BCL6* were detected. A positron emission tomography/computed tomography (PET/CT) showed a hypermetabolic uptake by multiple supra- and sub-diaphragmatic lymph nodes, spleen, left iliopsoas muscle and BM. The patient received 5 cycles of R-CHOP (rituximab, cyclophosphamide, doxorubicin, and vincristine) plus 1 cycle of high-dose methotrexate (as prophylaxis for central nervous system involvement) that only led to a partial remission (PR) at PET/CT scan. She then underwent two cycles of salvage chemotherapy with R-DHAOX (rituximab, cytarabine, and oxaliplatin) followed by collection of CD34+ peripheral hematopoietic stem cells. After a FEAM conditioning regimen (fotemustine, etoposide, cytarabine, and melphalan), she underwent an autologous hematopoietic stem cell transplantation (auto-HSCT) without significant response (**Figure 1A**). Therefore, the patient was regarded as eligible for CAR-T-cell therapy and underwent an apheresis collection of lymphocytes. We opted for sTLI as a bridge to CAR T cells instead of polatuzumab-based regimens because polatuzumab was not yet available from the Italian Drug Agency (AIFA). Moreover, the disease appeared chemorefractory and the patient was radiotherapy-naïve raising the opportunity to obtain a certain degree of response. In particular, 20 Gy was delivered in 10 fractions over 5 days in all PET/TC positive tumor sites (i.e., the left iliopsoas muscle and all the main nodal stations, minus the mediastinum) except for the spleen, which received 11.5 Gy (**Figures 1B, C**). One month later, the disease evolved to leukemia (WBC 3,900/μl [normal: 4,000–9,000/μl], 70% tumor lymphoid cells) and the patient became transfusion dependent due to marked anemia (Hb 7.9 gr/dl [normal: 13 to 17 g/dl]) and thrombocytopenia (platelets, 10,000/μl [normal: 140,000–400,000/μl]). A BM biopsy showed massive involvement by DLBCL expressing CD19 (**Figures 1D–G**). For this reason, the patient received TMI (18 Gy; 1.8 Gy × 2/die for 5 days) (**Figures 1H, I**), followed by thiotepa (5 mg/kg) and an infusion of residual, previously collected autologous CD34+ hematopoietic stem cells (5.5 × 10^6^/kg). Side effects included grade 4 mucositis limited to the mouth and requiring opioids, fever due to *Staphylococcus haemolyticus* sepsis (detected at blood cultures) that was successfully treated with daptomycin, and an asymptomatic increase of HHV6 copies in the peripheral blood for which she received ganciclovir.

**Figure 2:**
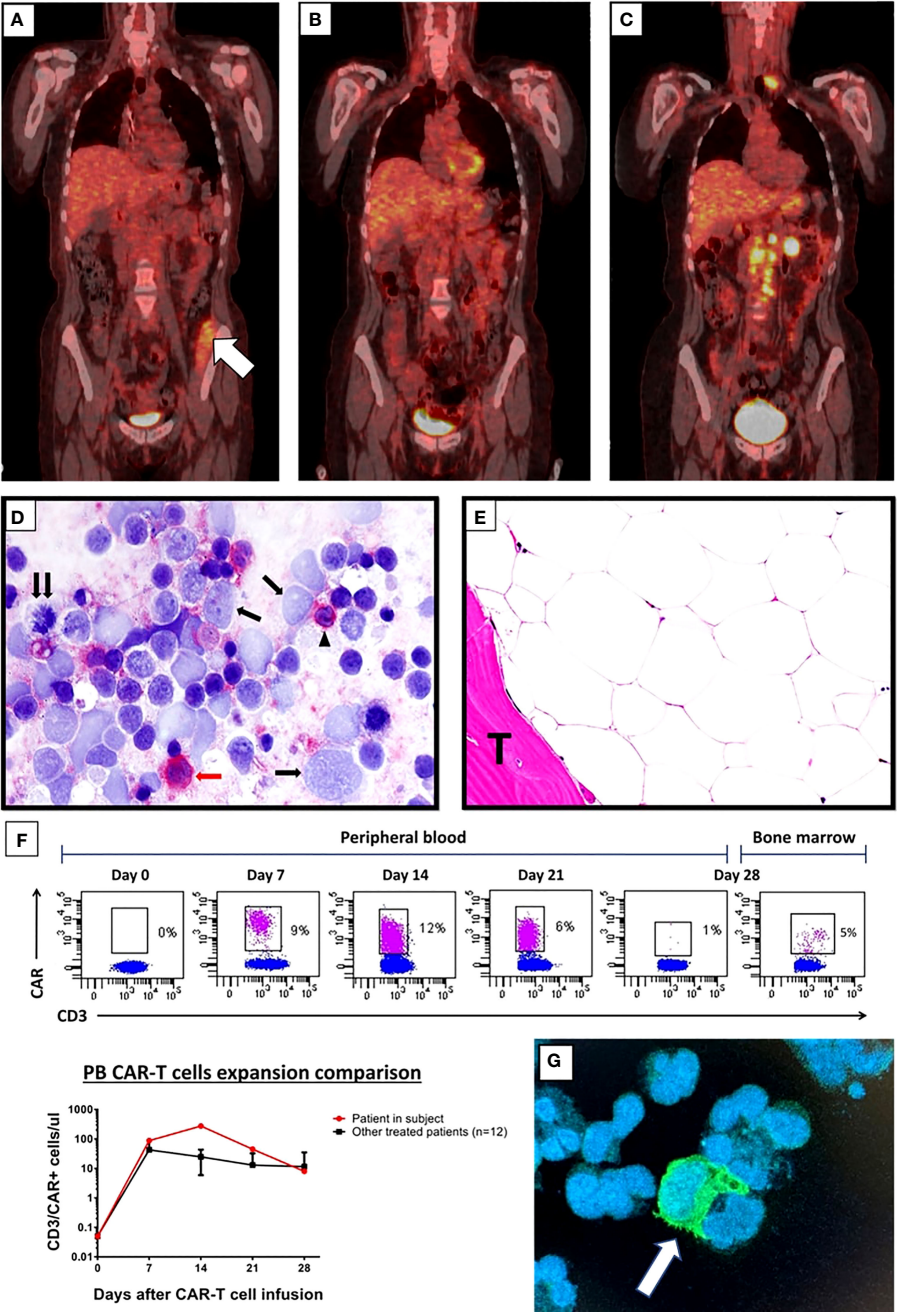
**(A–C)** FDG-PET/CT coronal maximum intensity projection (MIP) image after sTLI/TMI and before CART cells; white arrow in **(A)** indicates metabolic uptake in the iliopsoas muscle **(A)** FDG-PET/CT after CD19-directed CAR-T-cell therapy showing metabolic complete response at 6 months **(B)** and DLBCL relapse at 11 months **(C)**. **(D)** Imprint of latero-cervical lymph node at relapse immunostained for the CD19 CAR target (detected in red). Almost all large lymphoma cells appear CD19-negative (single arrows); the double arrow points to a CD19-negative tumor cell in mitosis. The red arrow indicates a CD19 positive (red) large tumor cell while the arrowhead indicates a CD19 positive (red) normal small B lymphocyte (Alkaline Phosphatase Anti-Alkaline Phosphatase (APAAP) technique; ×400). Negativity of >95% of tumor cells for CD19 was also confirmed in frozen and paraffin sections of the lymph node (not shown). **(E)** BM biopsy taken 11 months after CAR-T cell infusion showing a markedly hypocellular marrow without lymphoma infiltration. T indicated BM trabecula. (Hematoxylin–Eosin; ×400). **(F)** Flow cytometry plots showing CAR T cells detected in the CD3+ T-cell subsets in the peripheral blood every week the first month after CAR-T cell infusion and in bone marrow aspirate at day 28-disease assessment (top). Comparison of CAR-T cell absolute count expansion between the patient in subject and mean of the other treated patients in our center (n = 12) at indicated time points after CAR T-cell infusion (bottom). CAR T cells were detected staining anti-CD19 CARs by the biotinylated CD19 CAR detection reagent (Miltenyi) together with anti-biotin-APC. **(G)** Immunofluorescence image stained by biotinylated CD19 CAR detection reagent (Miltenyi) together with anti-FC FITCH conjugated secondary antibody (Thermo-Fisher, green) and DAPI (for cell nuclei, blue) performed on cytospin preparation from the peripheral blood of the patient obtained 14 days after CAR T-cell infusion. The white arrow indicates a CAR-T cell probably embracing a leukemic B cell.

After sTLI and TMI therapy, the PET/CT showed the disappearance of all metabolic positive lymphadenopathy but persistence of uptake in the left iliopsoas muscle (**Figure 2A**), while BM biopsy revealed about a 50% reduction of tumor cells. The residual lymphoma B cells expressed CD19 by immunohistochemistry. The blood cell count (BCC) showed: WBC 3,440/μl with the disappearance of circulating lymphoma cells, Hb 8.2 and an increase in platelet number (132,000/μl).

**Figure 3:**
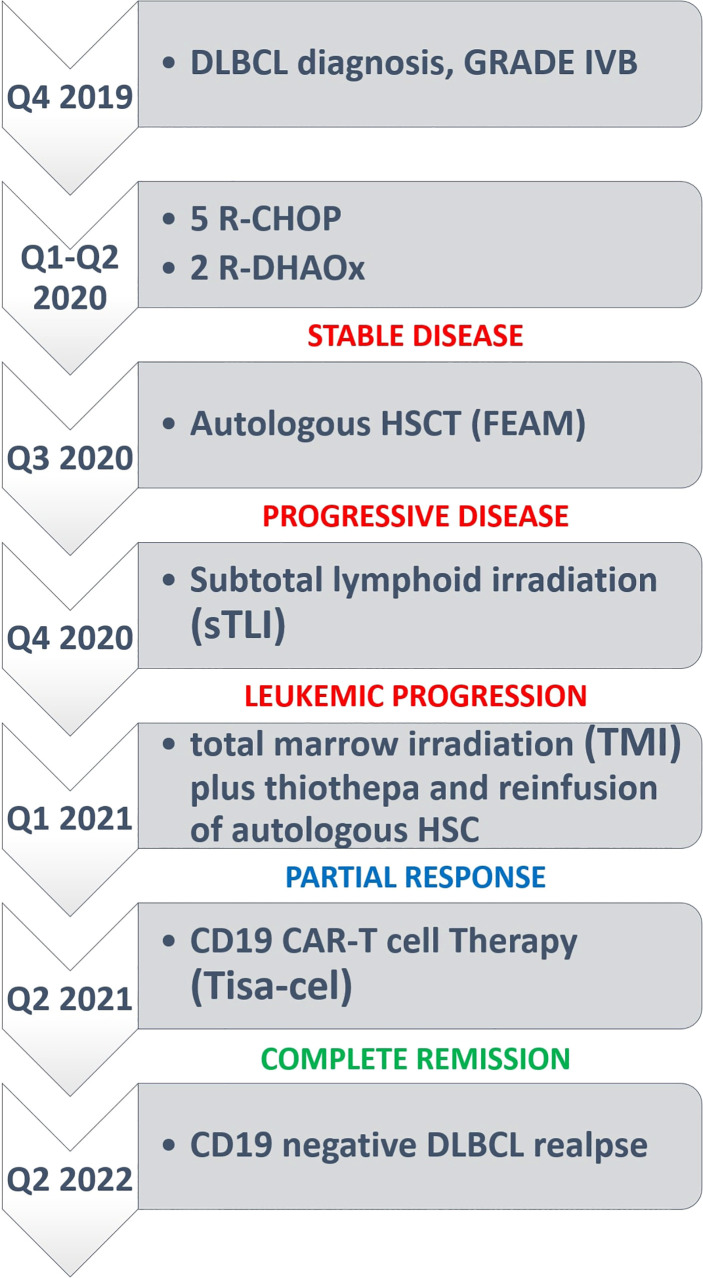
Timelines of events.

Given the good response to bridging therapy, the patient underwent lymphocyte depletion with fludarabine and cyclophosphamide, followed by a tisagenlecleucel infusion. She received a total of 130 × 10^6^ CD3+ cells, with a 33% CD19-CAR-transduced T cell and a 2:1 CD4:CD8 ratio. After CAR T cell infusion, she experienced a grade 3 cytokine release syndrome (CRS) characterized by fever and hypotension that was successfully treated with tocilizumab (four doses), single dose dexamethasone (20 mg), and supportive therapy. Indeed, PET/CT scans performed 1, 3, and 6 months after tisagenlecleucel showed CR (**Figure 2B**). A prolonged neutropenia was observed with BCC returning to normal at 6 months (WB 4.970/μl, N 45%, L 41%, M 10%, Hb 11 g/dl, PLT 274,000/μl). Notably, despite previous TMI, the patient did not experience prolonged anemia or thrombocytopenia after CAR-T-cell therapy. CAR T-cell expansion, monitored by flow cytometry every week after CAR T infusion, showed high CAR T cell levels in peripheral blood 14 days after infusion (276 CAR T positive cells/microliter) (**Figures 2F, G**). At 3 and 6 months after CAR T cell therapy, very low counts of normal B lymphocytes were detected by flow cytometry (B-cell aplasia), supporting the evidence of long-term CAR T cell persistence. At 11 months of follow-up, PET-CT showed a relapse in the left laterocervical and several retroperitoneal lymph nodes (**Figure 2C**) due to CD19 antigen escape (**Figure 2D**), while the BM biopsy showed a markedly hypocellular marrow without infiltration by lymphoma (**Figure 2E**). Because of her young age and good performance status, she is now being considered for haploidentical HSCT (**Figure 3**, timeline of events).

## Discussion and conclusions

Patients with R/R DLBCL frequently require bridge therapy to decrease the tumor burden before CAR T-cell infusion (10). In fact, low tumor burden has been associated with improved overall response rate, durability of response at 1 year (2, 11, 12) and lower treatment-related toxicity, mainly CRS (9). Moreover, an increased LDH (13) or a high metabolic tumor volume-MTV on PET/CT (14) in R/R DLBCL treated with axicabtagene, correlated with shorter PFS and OS. Similarly, a high tumor burden was predictive of lower event-free survival in adult B-ALL patients treated with CD19-directed CAR T cells (15). Thus, optimal tumor debulking before CAR T-cell infusion can potentially improve the outcome.

To our knowledge, this is the first time that sTLI and TMI have been used as bridging therapies to CAR T-cell infusion in a chemorefractory leukemic DLBCL. In general, RT appears particularly attractive as bridging therapy to CAR T cells, especially in patients with highly chemorefractory (10, 16–18) and high tumor burden (19). In one study, bridging RT was superior to bridging chemo-immunotherapy in terms of PFS (10), allowing all patients to receive CAR T cells (axicabtagene) versus 74 and 67% of patients who underwent other forms of bridging therapy. So far, bridging RT has been mainly delivered to limited target volumes, independently of disease extension (10). However, in the present patient, sTLI to all involved areas was used to reduce out-of-field disease progression (10, 20). Although CD19 CAR T cells can eradicate substantial tumor cell infiltration in B-ALL in progressive disease settings, data in DLBCL are limited. Our patient had a high-burden progressive disease (including massive BM and peripheral blood involvement) that in DLBCL has been associated with a lower response to CAR T cells and higher rates of CRS and ICANS. Thus, going ahead with CAR T cell therapy would have probably increased the risk of severe CAR-related toxicities. Based on these considerations, we decided to deliver TMI followed by an infusion of autologous CD34+ hematopoietic stem cells before CAR T cells. Slight B-cell lymphoma contamination of the CD34+ purified hematopoietic stem cells of the patient was disregarded because contaminated cells were expected to be killed by CAR T cells. Despite the fact that the treatment in our patient was very active resulting in a CR of almost one year duration, she unfortunately relapsed because of CD19 escape.

The optimal RT dose and fractionation schedule for CAR T cells remain unclear. Commonly used doses are 30 Gy (3 Gy fraction) or 20 Gy (4 Gy fraction), which have been associated with local control in about 80% of patients (8). In a retrospective assessment, diverse schedules (median total dose of 35 Gy in a median of 2.5 Gy fraction) had no impact on PFS (10). Large irradiated volumes in our patient dictated the fractionation schedule, which was derived from our conditioning regimen for haploidentical HSCT with regulatory and effector T cells in AML using TLI plus TMI (21). This “comprehensive” RT allowed bridging to CAR T cells and achieving almost 1 year of CR in an otherwise incurable case. TLI + TMI sculpts radiation doses to lymph nodes, spleen, and bones while reducing them to visceral organs (22, 23). Further clinical and laboratory assessments will help determine whether using this RT approach may be of benefit in patients with high burden disease and improving blood cell count, as in the present case. On the other hand, it remains unclear whether bridging “comprehensive” RT provides better outcomes than irradiating small volumes in candidates for CAR T cell therapy. In fact, the benefits of localized bridging RT may extend beyond the irradiated area by inducing systemic immune-mediated anti-tumor responses, the so-called abscopal effect (24). Local irradiation has been reported to sensitize tumor cells to adoptive T-cell therapy through a number of mechanisms (25–27), including: i) the release of tumor-associated antigens, facilitating their cross-presentation by dendritic cells and antigen-specific T-cell priming; ii) enhancing migration of cytotoxic T lymphocytes to irradiated areas *via* increased release of chemokines; and iii) improving their proliferation and effector function in irradiated sites. Robust CAR T-cell expansion and long-time persistence have been associated with enhanced responses and prolonged survival, while poor *in vivo* proliferation has been closely correlated with failure (28).

In conclusion, salvage treatments of R/R DLBCL are rapidly evolving with novel approaches such as bispecific and drug-conjugated antibodies, including polatuzumab combinations. In the near future, the main challenge will be to find the best bridge therapy for CAR T cells according to patient and disease features. Bridging therapy may not have only the role of controlling the disease during CAR T-cell manufacturing but should be part of the treatment, with the aim of further improving the expansion and persistence of adoptive T-cell therapy and, consequently, the outcome. For patients at increased risk of non response/relapse following CD19-directed CAR T-cell therapy, as the patient presented here, the role of bridging “comprehensive” radiotherapy, including TLI ± TMI approaches and potentially consolidative allogeneic SCT, should be further evaluated in clinical trials.

## Data Availability Statement

The original contributions presented in the study are included in the article/**Supplementary Material**. Further inquiries can be directed to the corresponding authors.

## Ethics Statement

Written informed consent was obtained from the individual(s) for the publication of any potentially identifiable images or data included in this article.

## Author Contributions

SS, VMP, CA, and BF conceived and designed the study. SS, CF, and CA carried out Radiotherapy. VMP, LFa, AI, SB, LFi, and BF managed the patient. VMP and BF carried out the pathological analysis. LR, SC, and AP carried out the immunophenotype analysis. BF wrote the manuscript. SS, VMP, LF, and CA approved the final draft of the manuscript. All authors listed have made a substantial, direct, and intellectual contribution to the work and approved it for publication.

## Conflict of Interest

The authors declare that the research was conducted in the absence of any commercial or financial relationships that could be construed as a potential conflict of interest.

## Publisher’s Note

All claims expressed in this article are solely those of the authors and do not necessarily represent those of their affiliated organizations, or those of the publisher, the editors and the reviewers. Any product that may be evaluated in this article, or claim that may be made by its manufacturer, is not guaranteed or endorsed by the publisher.
